# Economic growth, renewable energy and financial development in the CPTPP countries

**DOI:** 10.1371/journal.pone.0268631

**Published:** 2022-06-16

**Authors:** Duc Hong Vo, Quan Tran, Thao Tran

**Affiliations:** 1 Research Centre in Business, Economics & Resources, Open University, Ho Chi Minh City, Vietnam; 2 International School of Business, University of Economics, Ho Chi Minh City, Vietnam; Szechenyi Istvan University: Szechenyi Istvan Egyetem, HUNGARY

## Abstract

The trade agreement is generally considered an effective mechanism to encourage trading activities. However, trade activities may lead to environmental degradation because more trade is generally associated with more energy consumption. In addition, financial development with an increased flow of capital among members is required to fund trading activities. Renewable energy can be a moderating factor to balance the effects of trade activities and financial development on the economy and the environment. This paper focuses on the inter-relationship between growth-energy-finance nexus for the CPTPP members in the 1971–2020 period. While the energy-growth-environment nexus has been extensively investigated, the energy-growth-finance relationship has been largely ignored in existing literature, particularly for the CPTPP countries. Our findings can be summarized as follows. First, we find that renewable energy consumption does reduce CO_2_ emission while financial development does not necessarily increase environmental degradation. Second, financial development is found to cause renewable energy usage bilaterally. Finally, when different proxies are used for financial development, a bilateral causality relationship between renewable energy usage, financial development and economic growth is confirmed. These important findings imply that the governments of the CPTPP countries should encourage renewable energy usage to achieve the dual objectives from the CPTPP trade agreement: (i) to increase trade activities; and (ii) to support further financial development within the region. These two objectives together support economic growth.

## 1. Introduction

The trade agreement is widely seen as a catalyst for advancing global free trade and accelerating economic growth. Free trade agreements have increased from 19 in 1990 to 292 in 2019 [1]. A trade agreement is generally used to benefit countries through trade creation and market expansion by eliminating trade barriers and liberalizing foreign investment. They can also expand to different aspects, such as services, procurement, intellectual property rights, regulatory cooperation, and sustainable development. However, a growing concern about the prospect of increased trade could lead to environmental degradation. Countries with comparative advantages in products causing pollution may be incentivized to specialize in producing those products. Competition between countries can also lead to a “race to the bottom” phenomenon, where countries attempt to lower their environmental standards and regulations to shield domestic firms from international competition.

An appropriate balance between encouraging trade and minimizing environmental degradation has become an increasingly important topic among academics, practitioners and policymakers. Many scholars point towards renewable energy as the moderating factor between enhancing trade activities through exports for economic growth and improving environmental degradation by limiting CO_2_ emissions. Empirical studies have examined the relationship between economic growth and environmental degradation for different samples of countries, periods of study, and empirical techniques. However, the empirical results have been far from conclusive. [2] investigate the relationship between CO_2_ emissions, energy consumption and economic growth in ASEAN-5 over 1980–2016. Their findings confirm the unidirectional relationship between these important variables. [3] use the bootstrap-rolling-window estimation method to examine the growth-energy-environment nexus. They conclude that an inverted U-shaped environmental Kuznets curve is found for the US.

In contrast [4], investigate this growth-energy-environment nexus in Vietnam. Findings from this study indicate that no significant impact from export and renewable energy consumption on pollution is found for Vietnam. Other studies such as [5–7] produce mixed empirical results concerning this important growth-energy-environment nexus. We consider that mixed empirical results from previous studies can be explained by different designs of the studies, including the methods used in the analysis, together with different periods of study and different countries. Given the dynamic nature of economic growth, energy consumption, and environmental quality, a one-size-fits-all analysis appears impossible.

Our literature review indicates that a sample of countries in the recently established Comprehensive and Progressive Agreement for Trans-Pacific Partnership (or CPTPP) in 2018 has largely been ignored in investigating the inter-relationship between economic growth, energy consumption and environmental degradation. The Trans-Pacific Partnership (TPP) agreement had been initiated, discussed and signed among 12 countries in 2016. However, this TPP agreement never entered into force because the US withdrew from the agreement soon after a new government in 2017. The remaining 11 countries had put great effort to revive the agreement, known as the CPTPP agreement. The CPTPP has finally come into force in 2018.

This study contributes to the existing literature by examining the role of renewable energy in economic growth and financial development between the Trans-Pacific countries. The Comprehensive and Progressive Agreement for Trans-Pacific Partnership (CPTPP) is a free trade agreement between Australia, Brunei Darussalam, Canada, Chile, Japan, Malaysia, Mexico, Peru, New Zealand, Singapore and Vietnam. We note that the hoped-for benefits of the CPTPP agreement are multiple and diverse. For example, the CPTPP agreement has been considered the new gold standard for trade agreement in the context of digital trade and e-commerce, the importance of the small-medium enterprises (SMEs) and the promotion of civil society values. However, the scope of this analysis is much more limited. This analysis focuses on the inter-relationship between economic growth, renewable energy, and financial development and their causality relationship flows. In particular, we use seven proxies for financial development to ensure the robustness of the findings from this study. Among other countries, the United Kingdom and China have now applied to join the CPTPP agreement in 2021. It is noted that a significant difference in the per capita income across the members is observed. In addition, the differences in environmental standards and regulations among country members appear to be other significant concerns. As such, balancing the benefits to the national economies (through economic growth) and the environmental costs (due to further environmental degradation) arising from this trade agreement is considered essential. Therefore, considerations now focus on the role of renewable energy in these countries.

The higher-income countries are generally better at transitioning from coal and fossil fuel to renewable energy sources. For instance, in New Zealand, renewable energy accounts for 40 per cent of the country’s total energy consumption in 2019. In Australia, 24 per cent of electricity was generated by renewable energy, with over 50 per cent of South Australia’s energy coming from renewable sources. Canada and Japan also record relatively high renewable usage proportions at 17.3 and 18.5 per cent in the total energy mix [8, 9]. Another group of members involves middle-income countries that have successfully adopted renewable energy. Chile has achieved the highest growth of solar energy globally, with renewable energy accounting for 23.3 per cent of the country’s total energy generation. With sizeable crude oil and natural gas reserves, Mexico has generated 20.8 per cent of electricity from clean energy, of which 15.5 per cent comes from renewable energy [8, 9]. Finally, some CPTPP countries have been lagging in renewable energy consumption. For example, Singapore has to import almost all its energy needs because of the scarcity of natural resources. Brunei has only 0.05 per cent of its energy usage coming from solar energy since the country is highly rich in fossil fuel. Malaysia (2 per cent), Peru (2.7 per cent) and Vietnam’s (9 per cent) low renewable energy usage are mainly derived from the lack of comprehensive plans implemented by the governments of these countries [8, 9].

The contributions of this paper to the existing literature are threefold. *First*, the paper focuses exclusively on the trans-pacific region due to the recent establishment of the CPTPP trade agreement in 2018. Findings from the paper provide direct implications for policies in the region concerning the inter-relationship between economic growth, renewable energy and financial development. *Second*, while the fundamental relationship between economic growth and environmental degradation is examined, the benefits of enhancing financial development from trade agreements are also considered in this analysis. Financial development has largely been ignored in the growth-environment nexus, particularly for the CPTPP countries. An increase in trade activities from the agreement is expected to support economic growth. However, increased energy consumption is generally associated with further environmental degradation when energy mainly comes from fossil fuel sources. Financial development plays an important role in providing a basis for shifting energy consumption from fossil fuel to renewable sources. As such, we consider that the role of financial development should be considered in any growth-energy nexus. *Third*, the causality relationship between economic growth, renewable energy and financial development is also investigated in this paper. Findings from this causality analysis provide policy implications for countries in the region to balance and enhance the economic benefits from the trade agreement and simultaneously minimize the negative impacts on the environment.

The structure of this paper is as follows. Following this introduction, section 2 discusses related studies. Section 3 presents the research methodology and data. Empirical findings are then discussed in section 4. Finally, section 5 provides conclusions and policy implications.

## 2. Literature review

In recent years, the relationship between economic growth and environmental degradation has gained significant attention among scholars, focusing on the validity of the environmental Kuznets curve (EKC) hypothesis. This EKC hypothesis states that economic growth leads to environmental damage at a low level and then improves the quality of the environment after a specific threshold of economic growth is achieved. The hypothesis implies that environmental quality is negatively affected by economic growth at the early stage. However, at high-income levels, economic growth improves environmental quality. The relationship between economic growth and environmental degradation follows an inverted U-shaped relationship. The EKC hypothesis receives criticism from various scholars. [10] consider that the EKC hypothesis is an essentially empirical phenomenon. The author argues that most estimates of EKC models are not statistically robust. The author also considers that concentrations of selected local pollutants have declined in developed countries. However, many pollutants have increased the levels of emissions. As such, the drivers of changes in pollution cannot be identified. Other scholars are also skeptical about the validity of the EKC hypothesis [11–13]. On the empirical aspects, we note that findings from empirical studies on the validity of the EKC hypothesis are mixed.

On the supportive side [14], employ the autoregressive distributed lag (ARDL) model to investigate the validity of the EKC hypothesis in Malaysia over the period 1980–2008. The study confirms an inverted U-sharped relationship between income and environmental degradation in the long run but not in the short run. Their findings imply that the EKC is a long-run phenomenon. Similarly [15], investigate the inter-relationship among CO_2_ emission, economic growth, value-added agriculture and energy consumption for seven major emerging economies including China, India, Brazil, Mexico, Russia, Indonesia, and Turkey for the 1990–2014 period. The study supports the validity of the EKC hypothesis in the long run. Their findings also confirm a bidirectional causality relationship between non-renewable energy usage and CO_2_ emissions. Findings from [16] study failed to support the linear relationship between CO_2_ emissions, energy consumption, and economic growth in five ASEAN countries (including Indonesia, Malaysia, the Philippines, Singapore, and Thailand). Their findings confirm a nonlinear relationship among these variables. Furthermore, the study finds that environmental degradation initially increased with economic growth and decreased when per capita gross domestic product (GDP) was above 4,686 USD. Using a sample of 19 nations in the G20 group [17], confirm that agriculture increased CO_2_ emissions in developing countries, while renewable energy consumption reduces the CO_2_ emissions in developed countries. [3] utilize bootstrap rolling window estimation of vector autoregressive (VAR) model for the United States from 1966 to 2013. Findings from this study confirm that the effects of economic growth on CO_2_ emissions increased from 1982 to 1996 and decreased from 1996 to 2013. This finding is consistent with the U-shaped EKC hypothesis. [18] use the ARDL estimation technique to examine the validity of the EKC hypothesis in Vietnam over the 1974–2016 period. Findings from this study confirm the existence of the EKC hypothesis in the long run, while evidence on EKC is not found in the short run.

On the other hand, many empirical studies have provided evidence against the validity of the EKC hypothesis. For example [4], examine the causality link between CO_2_ emissions and economic growth in Vietnam for the 1981–2011 period. The results show that economic growth was positively related to pollution in both the short and long run. Therefore, economic growth is associated with pollution, proxied by CO_2_ emissions. These findings reject the validity of the EKC hypothesis in Vietnam. Also, capital is found to positively affect CO_2_ emissions. This finding indicates that an increased capital appears to be associated with the non-renewable-energy-intensive industries whereas the an increase in labour force may be linked with the emergence of lesser non-renewable-energy-intensive industries. Finally, we note that previous studies regarding the EKC hypothesis often assume a quadratic form between economic growth and CO_2_ emission.

In addition, findings from [5] analysis show that a non-parametric model confirms a positive relationship between CO_2_ emissions and economic activity without a turning point. These authors used a sample of the Middle-East and North Africa (MENA) countries from 1980 to 2010 and rejected the validity of the EKC hypothesis in these countries. Based on an ARDL framework, findings from [19] analysis reject the validity of the EKC hypothesis in Peru from 1980 to 2011 by comparing the long- and short-run elasticities of economic growth on the estimated coefficients of the CO_2_ emissions. The conclusion is drawn on the observation that the long-run estimated elasticity is greater than the estimated coefficient in the short run. Using the vector error-correction model (VECM) and data on Malaysia over the period 1975–2011 [6], show evidence against the validity of the EKC hypothesis. Finally [7], examine the nexus between renewable energy, growth and environmental quality for Canada and Australia over the 1960–2015 period. The paper’s main findings confirm that economic growth increases CO_2_ emissions in both the short run and long run and that renewable energy usage decreases emissions in the short run.

Also, mixed evidence is found regarding the validity of the EKC hypothesis in empirical studies. [20] examine the validity of the inverted U-shaped relationship between economic growth and environmental degradation using a sample of five ASEAN countries during the 1971–2009 period. The study utilizes the ARDL approach and Granger causality test on the VECM framework. Their results on the validity of the EKC hypothesis are mixed from this research sample. For example, the validity of the EKC hypothesis is confirmed in Singapore and Thailand but not in Indonesia, Malaysia and the Philippines. Empirical findings from their analysis confirm mixed results. [21] examines the relationship between renewable and non-renewable electricity consumption and economic growth for 26 OECD countries over 1980–2015. Using two different panel causality approaches, including the Dumitrescu-Hurlin time-domain approach and Croux and Reusens frequency domain approach, their analysis confirms the bidirectional relationship between economic growth and non-renewable electricity consumption.

As an interesting note, selected empirical studies suggest the N-shaped relationship between CO_2_ emissions and economic growth. [22] argue that different research samples, including the panel data and time-series analysis for individual countries, may yield different findings. The authors find supportive evidence for the EKC hypothesis using the panel mean group estimators for the sample of 20 Organization for Economic Cooperation and Development (OECD) countries. However, findings from only 9 out of 20 countries confirm the validity of the EKC hypothesis when each of these 20 OECD countries is considered, and six of them have a second turning point in the relationship between economic growth and CO_2_ emissions. An increase of CO_2_ emissions when income reaches the second turning point suggests an N-shaped rather than a U-shaped relationship between CO_2_ emissions and economic growth in these countries. The N-shaped relationship between CO_2_ emissions and economic growth is found in Vietnam in the long run. However, this relationship cannot be established in the short run [23].

Another strand of research emphasizes the causal relationship between CO_2_ emissions and economic growth. A seminal work by [24] reveals a causality from CO_2_ emissions to economic growth in the long run, without the feedback effects from economic growth to CO_2_ emissions in Malaysia for the 1971–1999 period. However, the result is only significant at a 10 per cent significance level. [26] confirm a bidirectional causality between CO_2_ emissions and economic growth in the short run in Malaysia over 1971–2015 when the VECM Granger causality test is used. Finally [27], investigate the effects of income on CO_2_ emissions for each of the five ASEAN countries, including Malaysia, the Philippines, Singapore, Thailand, and Indonesia using the Johansen cointegration test within the VECM framework. They find a bidirectional causality relationship between economic growth and CO_2_ emissions in Indonesia and Thailand in the long run. In the short run, a unidirectional causality from economic growth to CO_2_ emissions is also found for these two countries. Moreover, their results confirm a unidirectional relationship from economic growth to CO_2_ emissions in Malaysia and a bidirectional relationship in the Philippines and Singapore in the short run.

Our literature indicates that the relationship between economic growth, energy consumption, and environmental degradation has been extensively investigated in the existing literature. However, the inter-relationship between economic growth, renewable energy usage and financial development appears to be under-estimated, particularly for the CPTPP countries. Table 1 below presents a comprehensive summary of findings from papers examining the impacts and causal relationship between carbon emissions and economic growth and the EKC hypothesis and between carbon emissions, renewable energy, alternative and nuclear energy, and economic growth.

**Table 1:** Recent studies on carbon dioxide, economic growth, renewable energy, and alternative and nuclear energy.

Author	Sample	Period	Methodology	EKC	Variable	Short-run causality	Long-run causality
**Studies on carbon emissions and economic growth and the EKC hypothesis**							
[20]	5 ASEAN countries (Indonesia, Malaysia, Philippines, Singapore and Thailand)	1971–2009	Long-run estimates on ARDL models and VECM Granger causality	Yes	CO_2_, Y, Y^2^, EC	Y ↔ CO_2_ (Indonesia); CO_2_ → Y (Philippines); Y ↔ CO_2_ (Singapore); Y ↔ CO_2_ (Thailand)	Y ↔ CO_2_ (Indonesia, Malaysia and Philippines); Y → CO_2_ (Singapore and Thailand)
[4]	Vietnam	1981–2011	Short- and long-run estimates on ARDL models	No	CO_2_, Y, Y^2^, IMP, EXP, CA, L, ELF, ELR		
[28]	Top countries with REAI	1985–2011	Panel DOLS, panel FMOLS	Yes	CO_2_, Y, Y^2^, TO, FD, RE, NRE		
[17]	19 countries in G20	1990–2014	Panel FMOLC	Yes	CO_2_, Y, Y^2^, RE, Agri	RE ↔ CO_2_	RE ↔ CO_2_ Y ↔ CO_2_
[29]	Indonesia	1971–2010	Short- and long-run estimates on ARDL models	Yes	CO_2_, Y, Y^2^, RE		
[30]	Malaysia	1971–2012	Short- and long-run estimates on ARDL models, DOLS and VECM Granger causality	Yes	CO_2_, Y, Y^2^, TO, FD, FDI, EN	Y → CO_2_	Y → CO_2_
[31]	Pakistan	1971–2011	Short- and long-run estimates on ARDL models, Granger non-causality test	Yes	CO_2_, Y, Y^2^, TO, FD	Y → CO_2_	
[32]	25 emerging Asian countries	1980–2016	Panel FMOLS, DOLS, VECM causality and DH causality	Yes	CO_2_, Y, Y^2^, Y^3^, FDI, FF		Y ↔ CO_2_
[15]	E7 countries	1990–2014	OLS, Panel FMOLS, DOLS, VECM Granger causality	Yes	CO_2_, Y, Y^2^, RE, EN, Agri		EN ↔ CO_2_
[2]	5 ASEAN countries (Indonesia, Malaysia, Philippines, Singapore and Thailand)	1980–2016	Panel FMOLS, DOLS, Heterogeneous causality under cross-sectional dependence	Yes	CO_2_, Y, Y^2^, EC	Y → CO_2_ (Malaysia, the Philippines, Singapore, Thailand)	
**Studies on carbon emissions, renewable energy, alternative and nuclear energy, and economic growth**							
[33]	19 developing and developed countries	1984–2007	Panel ECM Granger causality	NA	CO_2_, Y, NU, RE		NU ↔ CO_2_, RE ↔ CO_2_, Y ↔ CO_2_
[34]	Tunisia	1990–2011	Short- and long-run estimates on ARDL models and VECM Granger causality	NA	CO_2_, Y, rail transport, maritime transport, RE		RE → CO_2_, Y → CO2
[21]	OECD	1980–2015	Dumitrescu-Hurlin panel causality test, Croux-Reusens panel frequency domain test	NA	Y, RE, NE		Y ↔ NE
[25]	Malaysia	1971–2015	ARDL, FMOLS, CCR long-run estimations and VECM causality	Inverted N-shaped	CO_2_, Y, Y^2^, Y^2^, RE	CO_2_ → RE, Y ↔ CO_2_	CO_2_ → RE, Y → RE
[35]	China and India	1965–2013	Short- and long-run estimates on ARDL models and VECM causality	Yes	CO_2_, Y, Y^2^, Hydro, UR		Y ↔ CO_2_
[36]	G7 countries	1991–2016	Panel FMOLS, DOLS, Fixed effect model, and Dumitrescu—Hurlin causality	Yes	CO_2_, Y, TO, RE	RE ↔ CO_2_, CO_2_ → Y	

*Note*: **AN**–consumption of nuclear and alternative energy; **CA**–capital; **CO**_**2**_ **–**CO_2_ emissions; **ELF**–electric consumption from fossil fuels, **ELR**–electric consumption from renewable resources; **EN**–energy consumption; **EN-rent**–total natural resource rents; **EXP**–the export; **FD**–financial development; **FDI**–foreign direct investment; **FF**–the consumption of fossil fuels; **HE**–the consumption of hydroelectric; **IMP**–the import; **IND**–the industrial value-added; **L**–labour; **NA**–Not available; **NRE**–the consumption of non-renewable energy; **NU**–Nuclear; **OP**–trade openness; **RE**–the consumption of renewable energy; **URB**–urbanization; **Y**–real per capita GDP; **Y**^**2**^ –the square of real per capita GDP.

An empty cell means that the causal relationship was not recorded.

Source: [26] and updated studies.

Our literature review highlights many important issues to the empirical investigation described below. These important issues are related to a careful examination of the growth-renewable energy-finance in the long run for the CPTPP countries. In addition, as presented in Table 1 below, many studies have been conducted to examine the growth-energy-environment nexus. However, it appears that countries joining the CPTPP trade agreement in 2018 have largely been neglected in these previous empirical analyses. This study is conducted to examine the inter-relationship between economic growth, renewable energy consumption, and financial development for the countries in the CPTPP agreement. In addition, our analysis also examines the causality relationship between these three aspects to provide empirical evidence on this important link.

## 3. Model and data

### 3.1. Model

The following model is used to examine the growth-energy-finance using a sample of the 11 countries from the CPTPP agreement. We select these variables in the regression equations based on empirical studies on the research topic [37–44]

$$CO_{2}{}_{it}=\alpha_{i}+\;\beta_{1i}GDPpc_{it}+\;\beta_{2i}GDPpc_{it}^{2}+\;\beta_{3i}EC_{it}+\;\beta_{4i}REC_{it}+\;\beta_{5i}FD_{it}+\;\varepsilon_{it}$$ (1)

where *CO*_*2*_ denotes CO2 emission, *GDPpc* represents economic growth, *GDP*^*2*^*pc* is a square of economic growth to examine the validity of the EKC hypothesis in these countries, *EC* denotes energy consumption, *REC* denotes renewable energy consumption, *FD* denotes financial development, and *ε* represents error term.

### 3.2. Data

Data are collected from the World Development Indicators (WDI) from the World Bank and International Monetary Fund (IMF). Our dataset consists of 11 CPTPP countries, namely Australia, Brazil, Canada, Chile, Japan, Mexico, Malaysia, New Zealand, Peru, Singapore, Vietnam. The 1970–2020 period is used in this paper. This research period presents the longest possible period to ensure the data availability required for our analysis. Details of variables are reported in Table 2. Descriptive statistics are presented in Table 3.

**Table 2:** Summary of variables.

Variable	Definition	Proxy	Source
***CO***_**2**_	CO_2_ emission	CO_2_ emissions (metric tons per capita)	WDI
***GDPpc***	Economic growth	GDP per capita (constant 2010 US$)	WDI
***EC***	Energy consumption	Energy use (kg of oil equivalent per capita)	WDI
***REC***	Renewable energy consumption	Renewable energy consumption (% of total final energy consumption)	WDI
***FD***	Financial development	New broad-based index of financial development	IMF

**Table 3:** Descriptive statistics.

	Observations	Mean	Median	Min	Max
*CO*_2_	257	8.102	7.305	0.301	25
Δ*CO*_2_	245	-0.005	0.043	-6.074	3.747
*GDPpc*	257	23,211	23,318	432	54,546
Δ*GDPpc*	245	413	302	-2,441	5,436
*EC*	257	3,421	3,568	260	9,829
Δ*EC*	245	24	18.551	-2,315	1,154
*REC*	257	466	195	1.014	1,870
Δ*REC*	245	2.413	0.913	-121	116
*FD*	257	0.518	0.526	0	0.952
Δ*FD*	245	0.009	0.009	-0.083	0.194

Note: CO_2_ denotes CO_2_ emission. GDPpc denotes economic growth. EC denotes energy consumption. REC denotes renewable energy consumption. FD denotes financial development. Δ denotes the first difference.

Descriptive statistics of the variables for our panel sample are shown in Table 2. For the CO_2_ emission, its highest level and lowest level are 25 and 0.301, respectively. The mean and median of the CO_2_ emission across these CPTPP countries are 8.102 and 7.305. On the first difference of the CO_2_ emission, its maximum value is 3.747 whereas the minimum value is -6.074. Economic growth has the highest mean value of 23,211 and a minimum mean of 432. The highest first difference in economic growth is 413 and a minimum of -2,441.

## 4. Estimation techniques

### 4.1. The cross-sectional dependence test

We employ the Pesaran CD test [45] to consider the cross-sectional dependence in this study. Ignoring cross-sectional dependence in the panel data analysis may produce inconsistent estimates. The CD statistics can be written as follows:

$$CD=\;\sqrt{\frac{2}{N\left(N-1\right)}}\overset{N-1}{\underset{i=1}{\sum }}\overset{N}{\underset{j=i+1}{\sum }}T_{ij}{\hat{\rho }}_{ij}^{2}\;\;\to \;N\left(0,1\right)$$

where ${\hat{\rho }}_{ij}^{2}$ denotes the correlation coefficients of residuals, N and T represent the cross-section and time dimensions. The CD statistics are asymptotically normally distributed if T and N go to infinity.

### 4.2. Slope homogeneity test

Besides the cross-sectional dependence, slope homogeneity is another issue of panel data. [46] note that the assumption of slope homogeneity would potentially ignore country-specific characteristics. As such, findings may provide a misleading conclusion. In this paper, we use the slope homogeneity test developed by [47]:

$$S=\;\overset{N}{\underset{i=1}{\sum }}{\left(\beta_{i}-\beta_{WFE}\right)}^{\prime }\frac{\chi_{i}^{\prime }M_{T}\chi_{i}}{{\tilde{\sigma }}_{i}^{2}}\left(\beta_{i}-\beta_{WFE}\right)$$

$$\Delta =\sqrt{N}\left(\frac{N^{-1}S-k}{\sqrt{2k}}\right)$$

where *S* and Δ are test statistics, *β*_*i*_ and *β*_*WFE*_ are estimates from the pooled ordinary least squares and the weighted fixed effect pooled estimator; *χ*_*i*_ denotes the matrix of independent variables in deviations from the mean; *M*_*T*_ is the identity matrix; ${\tilde{\sigma }}_{i}^{2}$ is an estimate of $\sigma_{i}^{2}$; k is the number of regressors. The bias-adjusted form Δ can be written as follows:

$$\Delta_{adj}=\sqrt{N}\;\left(\frac{N^{-1}S-k}{\sqrt{\frac{2k\left(T-k-1\right)}{T+1}}}\right)$$

### 4.3. Panel unit root test

We use the second-generation panel unit root test—the cross-sectional augmented Dickey-Fuller (CADF) developed by [48] to examine the stationarity of the considered variables. The CADF allows cross-sectional dependence. The test statistics are as follows:

$$CIPS=\;N^{-1}\overset{N}{\underset{i=1}{\sum }}CADF_{i}$$

where N is the number of panels. The *CADF*_*i*_ statistics are t-statistics from:

$$\Delta y_{it}=\;\alpha_{i}+\beta_{i}y_{it-1}+\theta_{i}{\bar{y}}_{t-1}+\sum_{j=0}^{p}\delta_{ij}\Delta {\bar{y}}_{t-j}+\sum_{j=0}^{p}\gamma_{ij}\Delta y_{it-j}+\varepsilon_{it}$$

where $\Delta {\bar{y}}_{t-j}$ and ${\bar{y}}_{t-j}$ are cross-sectional averages of first differences and lagged levels of *y*, respectively.

### 4.4. Panel cointegration test

Next, we use the panel cointegration test developed by [49] to examine the long-run equilibrium relationship among variables.


$$y_{it}=\;\alpha_{i}+\beta X_{it}\;+\;\varepsilon_{it}$$

$$\Delta y_{it}=c_{i}+\alpha_{i}\left(y_{it-1}\;-\;\beta_{i}x_{it-1}\right)+\overset{p}{\underset{k=1}{\sum }}\alpha_{1i}\Delta y_{it-k}+\overset{p}{\underset{m=1}{\sum }}\beta_{1i}\Delta x_{it-m}+\varepsilon_{it}$$

The augmented Dickey-Fuller test is obtained from the estimation of the following model:

$$\varepsilon_{it}=\theta \varepsilon_{it-1}+\overset{p}{\underset{k=1}{\sum }}\alpha_{k}\Delta {\hat{\varepsilon }}_{it-k}+v_{it}$$

where *θ* is selected to ensure that *v*_*it*_ is serially uncorrelated.

### 4.5. Panel Granger causality test

The [50] panel’s Granger causality test is employed to examine the direction of causality among the variables of interest. The method has an advantage as it considers cross-sectional dependence. The test involves the following regression:

$$y_{it}=\alpha_{i}+\overset{M}{\underset{m=1}{\sum }}\theta_{i}^{m}y_{it-m}+\overset{M}{\underset{m=1}{\sum }}\delta_{i}^{m}x_{it-m}+\varepsilon_{it}$$

where *θ*_*i*_ and *δ*_*i*_ are vectors of estimates of lagged dependent variables and explanatory variables. The Granger causality test involves test statistics *δ*_*i*_. The null hypothesis has no Granger causality, whereas the alternative hypothesis has at least one Granger causality. These hypotheses can be rewritten as follows:

$$H_{o}:\;\forall \;m:\;\delta_{i}^{m}=0$$

$$H_{\alpha }:\;\exists \;m:\delta_{i}^{m}\neq 0$$

## 5. Empirical findings

### 5.1. Empirical results from the cross-sectional dependence test

Using the [45, 51] CD test, this study examines cross-section dependence. The empirical results, shown in Table 4, confirm cross-section dependence, indicating that the estimators allowing cross-section dependence are suitable in this study.

**Table 4:** Results from Pesaran’s CD test concerning cross-sectional dependence.

	*CO*_2_	*GDPpc*	*GDPpc* ^2^	*EC*	*REC*	*FD*
CD test	7.929***	30.347***	31.417***	29.025***	-0.328	37.263***
p-value	0.000	0.000	0.000	0.000	0.743	0.000

Note:

*** significant at 1 per cent confidence level. The null hypothesis is of cross-section independence. CO_2_ denotes CO_2_ emission. GDPpc denotes economic growth. EC denotes energy consumption. REC denotes renewable energy consumption. FD denotes financial development.

### 5.2. Empirical results from the slope homogeneity test

In this section, we perform the slope homogeneity test. The empirical results, shown in Table 5, reveal that the null hypothesis of slope homogeneity is rejected by Δ and Δ_*adj*_ statistics. The empirical findings and those presented in Section 5.1 imply that the estimation techniques used in this study should allow both cross-sectional dependence and slope homogeneity.

**Table 5:** Empirical results from the slope homogeneity test.

	Statistics	
	Δ	Δ_*adj*_
Eq (1)	4.342*** (0.000)	6.074*** (0.000)

### 5.3. Empirical results from the panel unit root test

Using the unit root tests developed by [48], we examine the stationarity and the integration order of the variables used in this paper. Pesaran’s test is employed because it allows cross-sectional dependence. The empirical results reported in Table 6 reveal that all variables contain unit root at the level whereas their first differences are stationary. Overall, we find that variables used in our analysis are integrated at I (1). This finding indicates that a long-run equilibrium relationship between the employed variables may exist.

**Table 6:** Empirical results from panel unit root test.

Variable	Level		First Difference		Order of Integration
	Constant (1)	Constant & Trend (2)	Constant (3)	Constant & Trend (4)	
*CO*_2_	2.439 (0.993)	2.151 (0.984)	-9.959*** (0.000)	-10.048*** (0.000)	I (1)
*GDPpc*	2.355 (0.991)	0.150 (0.560)	-7.576*** (0.001)	-7.543*** (0.000)	I (1)
*GDPpc* ^2^	3.955 (1.000)	2.595 (0.995)	-6.159*** (0.000)	-6.819*** (0.000)	I (1)
*EC*	-0.991 (0.161)	0.973 (0.835)	-9.513*** (0.000)	-8.469*** (0.000)	I (1)
*REC*	1.944 (0.974)	1.928 (0.973)	-3.565*** (0.000)	-2.203*** (0.014)	I (1)
*FD*	-0.595 (0.276)	-0.870 (0.192)	-10.345*** (0.000)	-7.010*** (0.000)	I (1)

Note: The p-values are reported in parentheses. The Z[t-bar] is reported.

*** significant at 1% level. The null hypothesis assumes that all series are non-stationary. CO_2_ denotes CO_2_ emission. GDPpc denotes economic growth. EC denotes energy consumption. REC denotes renewable energy consumption. FD denotes financial development.

### 5.4. Empirical results from the panel cointegration test

We note that all employed variables are integrated at I (1). We employ a panel cointegration test to examine the long-run equilibrium relationship. The empirical results presented in Table 7 suggest that all panels are cointegrated. These findings imply a long-run equilibrium relationship between variables. This finding indicates that Granger causality should be considered in this study.

**Table 7:** Results of cointegration test.

	Eq 1
**Kao**	
Modified Dickey-Fuller test	-1.202*** (0.11)
Dickey-Fuller test	-2.106*** (0.01)
Augmented Dickey-Fuller test	-2.552*** (0.00)
Unadjusted modified Dickey-Fuller test	-4.049*** (0.00)
Unadjusted Dickey-Fuller test	3.506*** (0.00)

*** significant at 1 per cent level.

### 5.5. Empirical results for the energy-growth-finance relationship in the CPTPP countries

This section presents the empirical results using the common correlated effects mean group (CCEMG) and the augmented mean group (AMG). The CCEMG and AMG estimation techniques are appropriate in this study because these estimation techniques allow both cross-sectional dependence and slope heterogeneity in the panel data analysis. The estimates are shown in Table 8 below.

**Table 8:** Results for the energy-growth-finance inter-relationship in the CPTPP countries.

	CCEMG	AMG
*GDPpc*	0.0001 (0.044)	0.000 (0.215)
*GDPpc* ^2^	-1.47e-08 (0.109)	-1.15e-08 (0.185)
*EC*	0.002*** (0.00021)	0.001*** (0.0003)
*REC*	-0.004*** (0.0013)	-0.003** (0.0011)
*FD*	0.325 (0.830)	0.019 (0.985)
Number of observations	250	250
RMSE	0.1059	0.2028

Note:

** significant at 5 per cent level,

*** significant at 1 per cent level.

G denotes economic growth. CO_2_ denotes CO_2_ emission. GDPpc denotes economic growth. EC denotes energy consumption. REC denotes renewable energy consumption. FD denotes financial development. RMSE stands for root mean square error.

*First*, our empirical results cannot confirm the relationship between CO_2_ emission and economic growth during the research period for the CPTPP countries. No evidence supports the view that economic growth leads to environmental degradation in the CPTPP countries. The estimated coefficients of the GDP per capita (*GDPpc*) and the squared GDP per capita (*GDPpc*^*2*^) are statistically insignificant. These findings confirm that the validity of the EKC hypothesis cannot be confirmed in the CPTPP countries during the research period. This finding is consistent with findings from the [4] study. *Second*, our empirical finding indicates the estimated coefficients of the energy consumption (*EC*) are statistically significant at a one per cent level of significance in both estimation techniques. These findings indicate that an increase in energy consumption from fossil fuel sources is associated with increased CO_2_ emissions, leading to environmental degradation. This evidence is consistent with [20] study. *Third*, findings from this paper confirm that renewable energy consumption significantly reduces CO_2_ emissions because the estimated coefficients of renewable energy usage (*REC*) are negative and statistically significant at a one per cent level of significance under both estimation techniques used in our analysis. An increase in renewable energy usage leads to a reduction of CO2 emissions, leading to improved environmental quality. This finding implies that renewable energy usage can be considered a moderating factor to balance the benefits from the trade agreement via an increase in trade activities which supports economic growth and the costs to the environment by limiting CO_2_ emissions, which may lead to further environmental degradation. The finding is consistent under both estimation techniques. We note that this finding is consistent with [52, 53].

### 5.6. Results of Granger causality test

**Fig 1:**
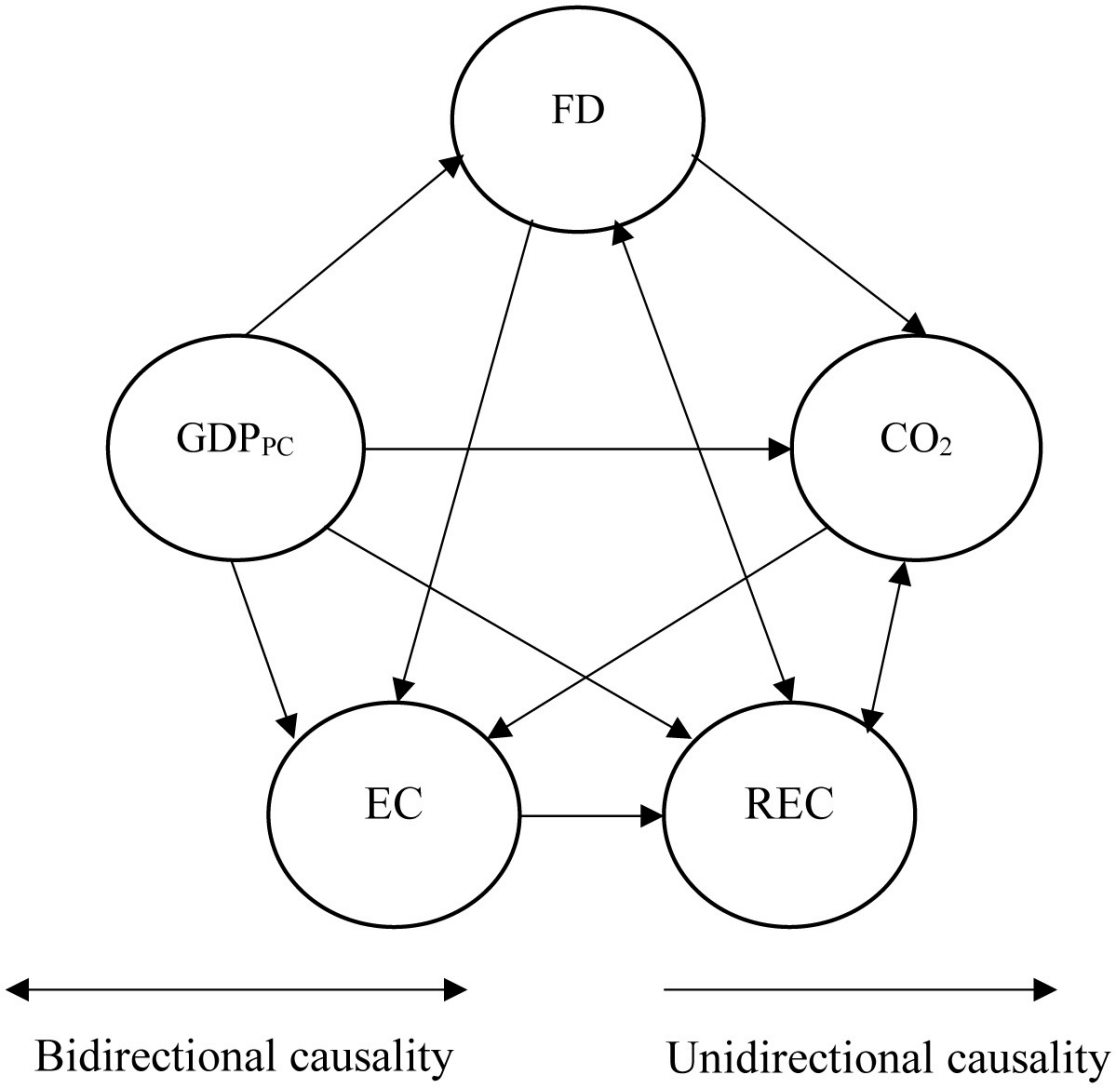
The causality relationship flows between economic growth (GDPpc), renewable energy usage (REC); energy consumption from fossil fuel sources (EC) and financial development (FD).

Next, we use the [50] panel’s Granger causality test to examine the relationship between economic growth, renewable energy usage and financial development. This analysis is important because the long-run relationships between these variables are confirmed in our analysis presented in section 5.4. Empirical results are presented in Table 9. We present these findings using the graphical causality relationship in Fig 1.

**Table 9:** The empirical results on the Granger causality relationship between economic growth, renewable energy usage and financial development in the CPTPP countries.

Hypothesis	Z-bar	Z-bar tilde	Conclusion
*GDPpc* → *CO*_2_	8.257*** (0.00)	7.444*** (0.00)	Unidirectional causality from economic growth to CO_2_ emission.
*CO*_2_ → *GDPpc*	1.557 (0.11)	1.230 (0.21)	
*EC* → *CO*_2_	7.616*** (0.00)	0.296 (0.76)	Bidirectional causality between energy consumption and CO_2_ emission.
*CO*_2_ → *EC*	6.460*** (0.00)	0.123 (0.90)	
*REC* → *CO*_2_	0.834 (0.40)	0.490 (0.62)	Unidirectional causality from CO_2_ emission to renewable energy consumption.
*CO*_2_ → *REC*	6.920*** (0.00)	5.566*** (0.03)	
*FD* → *CO*_2_	5.522*** (0.00)	4.757*** (0.00)	Unidirectional causality from financial development to CO_2_ emission.
*CO*_2_ → *FD*	0.074 (0.94)	-0.077 (0.93)	
*GDPpc* → *EC*	3.426*** (0.00)	3.051*** (0.00)	Unidirectional causality from economic growth to energy consumption.
*EC* → *GDPpc*	0.148 (0.88)	0.033(0.97)	
*GDPpc* → *REC*	1.757 (0.07)	1.281 (0.20)	Unidirectional causality from economic growth to renewable energy consumption.
*REC* → *GDPpc*	-0.522 (0.60)	-0.637 (0.52)	
*GDPpc* → *FD*	3.642*** (0.00)	0.215 (0.82)	Bidirectional causality between economic growth and financial development.
*FD* → *GDPpc*	8.352***(0.00)	1.720* (0.08)	
*EC* → *REC*	4.771*** (0.00)	3.818*** (0.00)	Bidirectional causality between energy consumption and renewable energy consumption.
*REC* → *EC*	3.692*** (0.00)	2.910*** (0.00)	
*FD* → *EC*	5.874*** (0.00)	0.929 (0.35)	Bidirectional causality between financial development and energy consumption.
*EC* → *FD*	6.078** (0.02)	0.994 (0.32)	
*FD* → *REC*	22.580*** (0.00)	18.807*** (0.00)	Unidirectional causality from financial development to renewable energy consumption.
*REC* → *FD*	1.394 (0.16)	0.976 (0.32)	

Note: A → B denotes unidirectional Granger causality running from A to B.

* significant at 10 per cent level,

** significant at 5 per cent level,

*** significant at 1 per cent level.

G denotes economic growth. CO_2_ denotes CO_2_ emission. GDPpc denotes economic growth. EC denotes energy consumption. REC denotes renewable energy consumption. FD denotes financial development.

### 5.7. A robustness analysis—The extended analysis using different proxies for financial development

The financial development index reported from the IMF has been used as a proxy for financial development in our analysis. However, financial development is a multifaced phenomenon, and previous analyses have used different financial development proxies [54, 55]. In this section, we use other indicators as the proxies for financial development. Our literature review indicates that each of the following seven indicators has been used as a proxy for financial development in previous studies, including (i) liquid liabilities to GDP; (ii) deposit money bank assets to GDP; (iii) private credit by deposit money banks to GDP; (iv) financial system deposits to GDP; (v) stock market capitalization to GDP; (vi) stock market total value traded to GDP, and (vii) stock market turnover ratio.

As a result, in this robustness analysis, we re-estimate the Eq (1) using the CCEMG and the AMG estimation techniques for each of these seven proxies for financial development to enhance the robustness of our empirical findings. Table 10 presents the summary of statistics for seven proxies of financial development.

**Table 10:** Descriptive statistics for seven proxies for financial development.

	Observations	Mean	Median	Min	Max
LLGDP	246	80.523	74.466	9.880	231.312
DBAGPP	246	89.091	87.682	6.226	241.548
PCRDBGDP	246	76.333	78.012	3.365	192.100
FDGDP	246	73.454	68.819	2.931	219.797
STMKTCAPGDP	236	83.549	76.180	0.408	265.563
STVALTRADEDGDP	236	36.140	20.948	0.079	173.804
STTURNOVER	236	41.270	32.379	1.574	165.080

Note: **LLGDP** denotes liquid liabilities to GDP. **DBAGPP** denotes deposit money bank assets to GDP. **PCRDBGDP** denotes private credit by deposit money banks to GDP. **FDGDP** denotes financial system deposits to GDP. **STMKTCAPGDP** denotes stock market capitalization to GDP. **STVALTRADEDGDP** denotes stock market total value traded to GDP. **STTURNOVER** denotes the stock market turnover ratio

Our empirical findings on the inter-relationship between economic growth, renewable energy usage and financial development are presented in Table 11 below. Our empirical findings fail to confirm the validity of the EKC hypothesis in CPTPP countries across seven proxies for financial development. This finding concerning the validity of the EKC hypothesis in the CPTPP countries is consistent with our previous finding. Findings from our robustness analyses confirm a positive relationship between energy consumption and CO_2_ emissions in the CPTPP countries across all seven proxies for financial development. These findings imply that any increase in energy consumption due to an increase in economic activities for producing products and providing services is associated with an increase in CO_2_ emission, leading to an increased environmental degradation in the CPTPP countries. This finding using different proxies of financial development is consistent with our conclusion on the effect of energy consumption from fossil fuel sources on CO_2_ emission in previous analysis when the broad-based new financial development index from the IMF is used. Interestingly, renewable energy consumption is negatively and statistically significant to CO_2_ emissions. This finding is consistent across seven different proxies of financial development, together with the IMF’s broad-based financial development index, which is used in our previous analysis. Finally, we find no evidence to confirm an impact of financial development, regardless of the proxies, on CO_2_ emissions for the CPTPP countries during the researched period. This finding is also consistent with our previous analysis when the broad-based financial development index is used.

**Table 11:** Results of the robustness checks using seven alternative proxies for financial development.

	Liquid liabilities to GDP		Deposit money bank assets to GDP		Private credit by deposit money banks to GDP		Financial system deposits to GDP		Stock market capitalization to GDP		Stock market total value traded to GDP		Stock market turnover ratio	
	CCEMG	AMG	CCEMG	AMG	CCEMG	AMG	CCEMG	AMG	CCEMG	AMG	CCEMG	AMG	CCEMG	AMG
*GDPpc*	0.000* (0.092)	0.000 (0.218)	0.000 (0.067)	0.000 (0.292)	0.000 (0.103)	0.000 (0.293)	0.000*** (0.000)	0.000 (0.190)	0.000 (0.284)	0.000 (0.829)	0.000 (0.881)	0.000 (0.905)	0.000** (0.020)	0.000 (0.278)
*GDPpc* ^2^	-2.50e-08 (0.184)	-1.68e-08 (0.221)	-2.54e-08 (0.166)	-1.41e-08 (0.162)	-3.56e-09 (0.691)	-9.77e-09 (0.260)	-9.69e-09 (0.307)	-2.27e-08* (0.095)	2.34e-09 (0.752)	3.05e-09 (0.655)	7.46e-09 (0.552)	-1.73e-08 (0.232)	-7.22e-09 (0.361)	-1.80e-08 (0.105)
*EC*	0.002*** (0.000)	0.001*** (0.000)	0.002*** (0.000)	0.001*** (0.000)	0.002*** (0.000)	0.001*** (0.000)	0.002*** (0.000)	0.001*** (0.000)	0.002*** (0.000)	0.002*** (0.000)	0.002*** (0.000)	0.002*** (0.000)	0.002*** (0.000)	0.001*** (0.000)
*REC*	-0.002*** (0.002)	-0.003*** (0.000)	-0.002*** (0.006)	-0.003*** (0.000)	-0.003*** (0.000)	-0.002*** (0.000)	-0.002*** (0.000)	-0.003*** (0.000)	-0.004*** (0.001)	-0.003** (0.016)	-0.004*** (0.001)	-0.002* (0.071)	-0.004*** (0.001)	-0.003*** (0.000)
*FD*	-0.000 (0.792)	-0.003 (0.393)	-0.000 (0.929)	-0.002 (0.612)	0.000 (0.993)	-0.004 (0.349)	0.004 (0.307)	-0.003 (0.553)	-0.000 (0.925)	0.000 (0.916)	0.004 (0.267)	0.004 (0.214)	-0.001 (0.645)	0.002 (0.402)
No. Obs.	242	242	242	242	242	242	242	242	236	236	236	236	236	236
RMSE	0.1281	0.2261	0.1227	0.2074	0.1243	0.2129	0.1257	0.2240	0.1257	0.2732	0.1353	0.2923	0.1257	0.2829

Note:

* significant at 10 per cent significant level,

** significant at 5 per cent significance level,

*** significant at 1 per cent significance level.

G denotes economic growth. CO2 denotes CO2 emission. GDPpc denotes economic growth. EC denotes energy consumption. REC denotes renewable energy consumption. FD denotes financial development. RMSE stands for root mean square error.

**Fig 2:**
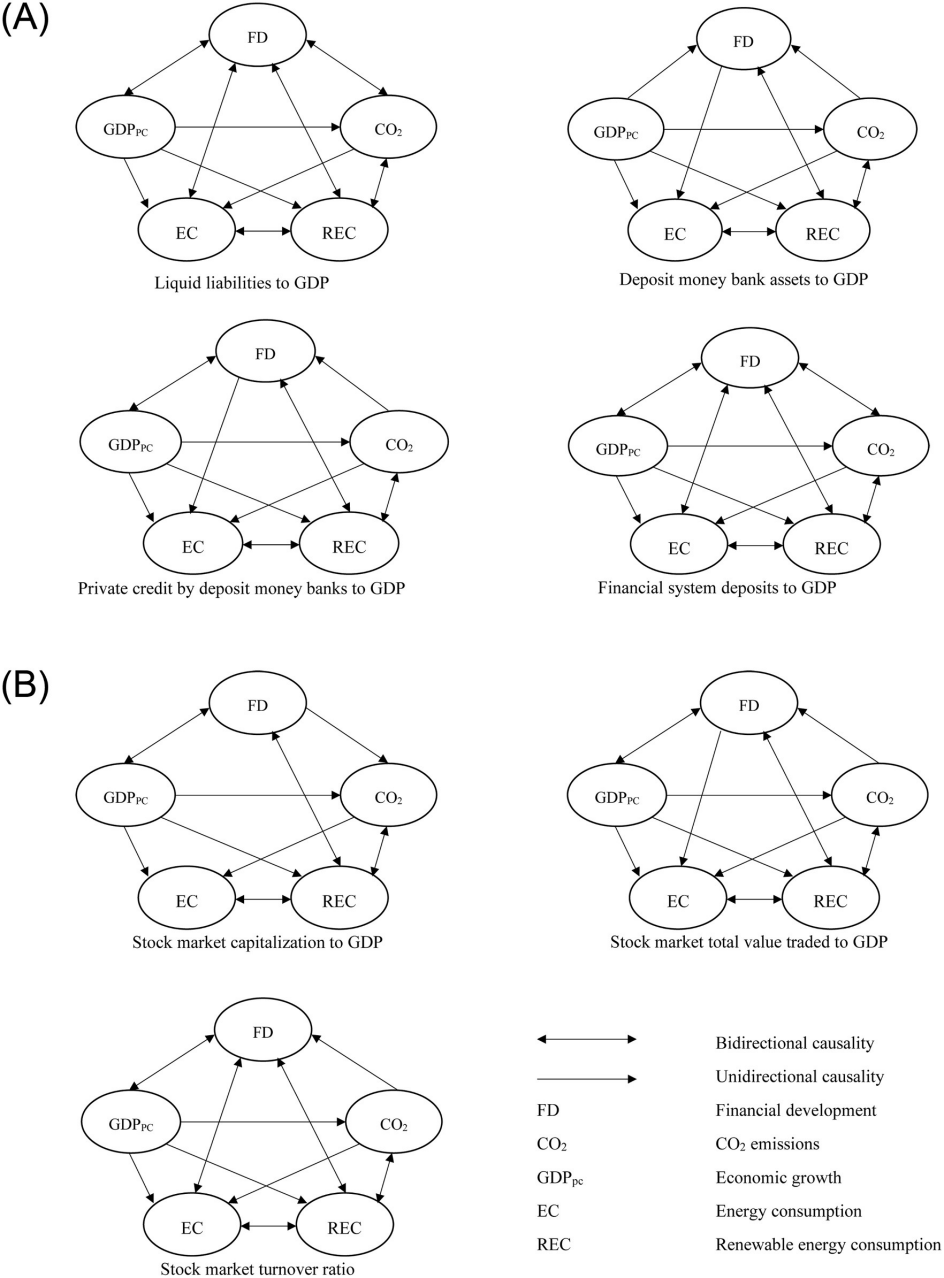
The causality relationship flows between renewable energy usage—Economic growth—Financial development using seven proxies.

In addition, our robustness analysis is extended by considering the causality relationship between renewable energy usage, economic growth and each of seven proxies for financial development for the CPTPP countries. Fig 2 below graphically presents these causality relationship flows.

## 6. Conclusions and policy implications

The Comprehensive and Progressive Agreement for Trans-Pacific Partnership (CPTPP), formerly the Trans-Pacific Partnership (TPP), agreement of 11 countries, came into force in 2018 after the US withdrew in 2017 under a new government. The agreement has attracted great attention from other countries to join the pact, including the UK and China, in 2021. Implementing the CPTPP agreement has been considered a milestone in economic integration across 11 country members in different continents. Trade agreements are generally considered an effective mechanism to increase trade flows across members. Country members from this mega CPTPP agreement will benefit from improved comparative advantages of producing products and providing services. However, increasing trading activities may increase energy consumption and CO_2_ emissions, leading to environmental degradation. In addition, an increase in trading activities may result in a further increase in financial development, which is required to support an increase in trading activities among the members. In order to achieve the dual benefits of trade agreements, the role of renewable energy in the national energy portfolio needs to be considered. We consider that renewable energy usage should be considered the moderating factor between enhancing trading activities, which support economic growth and financial development and improving environmental degradation by limiting CO_2_ emissions from the CPTPP agreement.

This paper examines the effects of renewable energy usage, financial development and economic growth on the environment for the countries joining the CPTPP agreement. Various proxies of financial development are used in this paper to enhance the robustness of the findings. Findings from this paper can be summarized as follows. *First*, renewable energy usage does reduce CO_2_ emissions in the CPTPP countries. This finding supports the view that increasing the proportion of renewable energy in the national energy portfolio does improve the quality of the environment by reducing CO_2_ emissions. This finding is consistent across different proxies of financial development used in our analysis. *Second*, energy consumption from fossil fuel sources does lead to an increase in CO_2_ emissions. This finding implies that reducing the energy usage from fossil fuel sources is important in improving the quality of the environment. Renewable energy can be considered excellent energy sources to substitute fossil fuel sources to ensure that energy is sufficient to support economic growth and development in the CPTPP countries. *Third*, no evidence indicates that further financial development, which is required to support the increase in trade activities from the CPTPP agreement, leads to further environmental degradation. This finding implies that further financial development to support economic growth and development will not negatively affect the environmental quality in the CPTPP countries. In addition, our causality analysis demonstrates that the bilateral causality relationship between renewable energy usage, financial development and economic growth does exist in the CPTPP countries. This important finding emphasizes the importance of considering the combined effects of renewable energy usage, financial development and economic growth.

Policy implications have emerged based on these important findings from this paper for countries in the CPTPP agreement and other markets. The success of the CPTPP agreement in achieving the overall goal for the Trans-Pacific region appears to be impossible without the support of each country member. Each CPTPP country should have a solid commitment to sustainable development. *First*, each government in the Trans-Pacific region needs to play an active role in improving the quality of the environment. In using trade agreements to support economic growth, renewable energy appears to be a solution for these countries to achieve fundamental objectives of (i) sustainable economic growth and development and (ii) improved quality of the environment. An increase in trading activities from the CPTPP agreement cannot ignore the support from financial development. We consider that ensuring the financial system operate efficiently, implementing friendly-environmental regulations, and encouraging green investment in potential industries are the responsibility of each government in the CPTPP region. The role of the development of the financial system in supporting economic growth and in reducing pollutant emissions has been confirmed in this analysis. *Second*, an emphasis on microeconomic perspectives is of equal importance. Investing in green investment in such sectors like renewables, eco-innovations, and bio-products should be encouraged to attain sustainable growth. An efficient financial system also helps the transition towards green investment smoothly. Doing so will enhance policy effectiveness in institutional development, organizational culture, and market efficiency for the economic growth—renewable energy usage—financial development inter-relationship. *Third*, raising awareness of the importance of using energy-saving products and adopting renewable energy among individuals, households, and firms will help mitigate the level of pollutant emissions, leading to improved environmental quality.

Our study suffers limitations. The CPTPP agreement is a game-changer for many countries around the world. The expected benefits from this mega agreement are significant and diverse. Our analysis focuses only on the inter-relationship between three macroeconomic issues, including economic growth, renewable energy usage, and financial development. Future studies may need to consider other issues from this mega-trade agreement for many countries globally, including analyses on digital trade and e-commerce and the important roles of SMEs in economic growth and development in these CPTPP countries.

## Supporting information

S1 Data (XLSX)
