# Correction: Economic growth, renewable energy and financial development in the CPTPP countries

**DOI:** 10.1371/journal.pone.0314387

**Published:** 2024-11-21

**Authors:** The PLOS ONE Staff

There is an error in affiliation 1 for author Duc Hong Vo. The correct affiliation 1 is: Research Centre in Business, Economics & Resources, Ho Chi Minh City Open University, Vietnam. The publisher apologizes for the error.
